# Lifestyle factors and co-morbidities associated with obesity and overweight in Nkonkobe Municipality of the Eastern Cape, South Africa

**DOI:** 10.1186/s41043-017-0098-9

**Published:** 2017-05-25

**Authors:** Wilfred Otang-Mbeng, Gloria Aderonke Otunola, Anthony Jide Afolayan

**Affiliations:** Medicinal Plants and Economic Development (MPED) Research Centre, Botany Department, University of Fort Hare (Alice Campus), Alice, 5700 South Africa

**Keywords:** Co-morbidities, BMI, Lifestyle factors, Nkonkobe Municipality, Obesity, Overweight

## Abstract

**Background:**

Obesity is a global epidemic that affects 500 million people worldwide and is predicted to increase to one billion people by 2030. The prevalence of obesity is increasing across populations in South Africa. However, questions still remain surrounding the predisposing factors and obesity-related health problems especially in the rural areas. This study evaluated several lifestyle factors such as dietary habits, physical activity, smoking, alcohol intake, co-morbidities and their association with the prevalence of obesity and overweight in Nkonkobe Municipality of the Eastern Cape.

**Methods:**

A cross-sectional, population-based survey was conducted among 118 residents in four rural/sub-urban townships of the study area. Measurements including weight, height, body mass index (BMI), physical activity and dietary habits were determined using a validated questionnaire.

**Results:**

The overall prevalence of obesity and overweight was 38 and 19%, respectively. The highest prevalence of obesity (70%) was observed among those who do not undertake any physical activity. Close to half (48.48%) of the respondents who eat fast foods always were obese, and 30.30% were overweight; when combined, the prevalence for obesity is 78.78%. A negative association with obesity was observed among regular smokers (26.92%) and consumers of alcohol (4.00%). Arthritis, hypertension and tuberculosis were co-morbidities significantly (*P* < 0.05) associated with obesity in the study area.

**Conclusions:**

The findings of this study reveal that lack of physical activity, overindulgence on fast and fried foods, low fruit and vegetable consumption as well as arthritis, hypertension and tuberculosis were significant risk factors of obesity in Nkonkobe Municipality.

## Background

Obesity is prevalent in most low- and middle-income countries and is associated with increasing cardiovascular disease (CVD) risk and health-related complications. In South Africa, the prevalence of overweight and obesity has increased steadily over time, reaching 56% in 2002 and 65% in 2012 with black African women living in urban townships and some rural communities as the most affected [1, 2].

According to Joubert et al. [3] and Okop et al. [4], obesity was the cause of 78% of type 2 diabetes, 68% of hypertensive disease, 45% of ischaemic stroke, and 38% of ischaemic heart disease cases among adults in SA. Information on the factors associated with overweight and obesity in black African women and men living in rural and urban communities are of critical importance for the development of community-specific obesity prevention strategies in these settings.

Current evidence indicates that obesity is a multifactorial condition influenced by many variables which include genetic, demographic and lifestyle factors. Al-Hazzaa et al. [5] posited that genetic and demographic variables such as family history of obesity, age, ethnicity and sex cannot be modified, but obesity-associated lifestyle factors such as physical inactivity, unhealthy dietary choices, smoking and alcohol intake are often modifiable.

Physical activity and fitness are important factors in reducing the risk of unhealthy weight gain and related illnesses. High levels of physical activity may protect against excessive weight gain whereas low levels permit excessive weight gain. Certain types of foods and eating habits such as fast foods, fried foods, snacking, binge-eating and eating out have been linked to weight gain and obesity [6].

The relationship between smoking and obesity is complex and not completely understood. While some studies have shown no significant association between smoking status and body mass index (BMI), others have reported that smokers tend to have lower BMI [7–9].

Although alcohol has comparatively high energy content, a controversy still exists on whether moderate amounts of alcohol represent a risk factor for weight gain and obesity. Several reports have shown positive, negative or no relationship between alcohol intake and body weight [10–13].

Overweight and obese persons are at an increased risk for developing many medical problems, including insulin resistance and type 2 diabetes mellitus, hypertension, dyslipidemia, cardiovascular disease, stroke, sleep apnoea, gallbladder disease, gout, osteoarthritis and cancer [14–16]. Reports have positively associated obesity with hypertension, diabetes, arthritis, and angina in India, China, Russia and South Africa [17]. There is convincing evidence that regular physical activity helps prevent unhealthy weight gain whereas sedentary lifestyles, particularly sedentary occupations and inactive recreational activities such as watching television, promote it [18, 19]. Traditionally, it has been thought that a high level of physical exercise could in part explain the low levels of chronic diseases found in sub-Saharan African countries, though the amounts of physical exercise have been decreasing as a result of high degree of urbanization [18].

The paucity of data on lifestyle factors and co-morbidities associated with obesity as well as the influence of such factors on obesity in sub-Saharan Africa raises the need for research among sub-urban and rural populations in order to develop effective and population-based interventions for the prevention of obesity. The objective of this study, therefore, was to determine the lifestyle factors and co-morbidities associated with obesity in Nkonkobe Municipality of the Eastern Cape, South Africa.

### Study Area

**Fig. 1:**
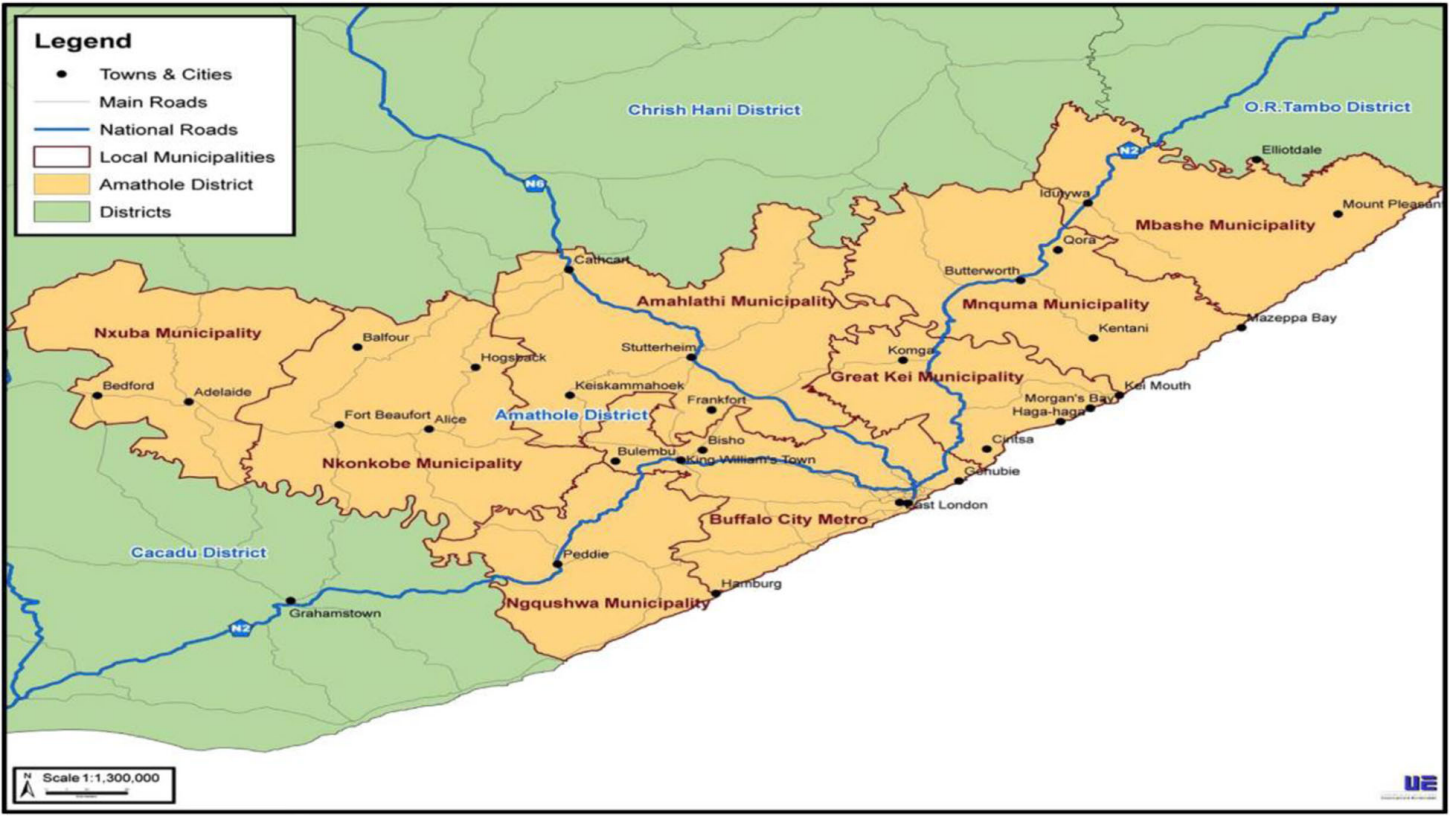
Map of Amathole District showing the Nkonkobe Municipality. Source: Otang et al. [20]

This study was carried out in the Nkonkobe Municipality of the Amathole District, Eastern Cape Province, South Africa, which falls within the latitudes 30° 00′ to 34° 15′ S and longitudes 22° 45′ to 30° 15′ E (Fig. 1).

The Nkonkobe Municipality is situated within the Amathole District Municipality of the Eastern Cape Province, between Port Alfred and Port St John. The climate is highly varied; the west is dry with sparse rain during winter or summer, with frosty winters and hot summers. The mean maximum temperature in January has been recorded as 26 °C and the mean minimum as 10 °C in July. The main tribes of the area are Xhosa-speaking peoples who are divided into several tribes with related but distinct heritages [20].

According to Statistics South Africa [21] (2015), Nkonkobe is comprised of 21 wards with a population of approximately 127,115 of which the majority (72%) resides in rural villages and farms. Nkonkobe is a rural municipality, and the economy is largely driven by the agricultural sector, which includes citrus, forestry and crop production. Some of the major challenges facing Nkonkobe Local Municipality include infrastructure challenges and high levels of unemployment and poverty.

## Methods

A population-based cross-sectional survey among 118 residents using weight, height, body mass index (BMI) and questionnaires was conducted in Alice, Fort Beaufort, Middledrift and Seymour of the Nkonkobe Municipality, from September to November 2015. Pairs of trained research assistants served as interviewers and a trained supervisor monitored the process in each location. The team members were given a day’s training of approximately 5 h in which each point of the questionnaire was explained and discussed. Special emphasis was given to the method of recording and measurement of height and weight. Practical demonstrations were also made for these parameters to avoid error during recording. Before the data collection began, the interviewers thoroughly explained to subjects the purpose and procedure of the study and obtained their consent to participate.

### Inclusion/exclusion criteria

Adults of both gender aged 21–70 years were eligible for inclusion in this study. Pregnant women, mothers who were less than 2 months post delivery and women who were on hormonal contraception, as well as subjects with oedema or wasting syndrome were excluded.

### Anthropometric measurements

Height and weight were measured with subjects in light clothes and without shoes using standard apparatus. Weight was measured to the nearest 0.1 kg on a calibrated beam scale. Height was measured to the nearest 0.5 cm with a non-expanding measuring tape.

### Questionnaire

The questionnaire collected data on physical activity, eating, smoking and alcohol habits of participants. Participants’ height and weight were obtained, and body mass index (BMI) was calculated as weight (kg)/height (m^2^). Physical activities were separated according to their intensity, which is defined as the distinction between walking, other moderate and vigorous physical activities for at least 10 min/day. Moderate activities are those that cause a small increase in respiratory frequency and require moderate physical exertion (such as carrying light loads and doing house chores) for at least 10 min each day; vigorous activities are those that cause more rapid breathing than normal, with considerable physical exertion (such as heavy lifting, digging and climbing stairs) for at least 10 min each day.

Overweight was defined as BMI equal to or more than 25 kg/m^2^, while obesity was defined as BMI equal to or more than 30 kg/m^2^. The presence of co-morbidities was determined by directly asking participants whether a physician had ever told them that they had a specific type of disease or health problem, and when the particular health condition had last been present.

### Ethical issues

Ethical clearance (UHREC/OTU001) was obtained from the Research Ethics Committee, University of Fort Hare. An informed consent to participate into the study was obtained from all participants prior to the study. Confidentiality of information was adhered to by using unique identification numbers on the data collection tools instead of participant’s names. Questionnaires were stored in locked cabinets at Medicinal Plants and Economic Development-Research Niche Area (MPED-RNA) and were accessible to the researchers only.

### Statistical analysis

The chi-square test (for categorical variables) and the Student *t* test (for continuous variables) were used to determine the lifestyle factors that were significantly (*P* < 0.05) associated with obesity.

## Results

The sample comprised of 118 participants, 64 females (54.24%) and 54 males (45.76%), and the ages ranged from 21 to 70 years. The overall prevalence of obesity and overweight in this representative sample was 38 and 19%, respectively

### Physical activity

For physical activity, participants were divided into three groups—no physical activity and moderate and vigorous physical activity—in order to assess the effect of lack of physical activity as a risk factor of obesity (Table 1). The highest prevalence of obesity (70%) was observed among those who were not physically active or consistent with exercise. The overall rates of obesity in the moderate and vigorous physical activity groups were 15.5 and 10.5%, respectively, and this difference was statistically significant (*P* < 0.05). Those who spent more days per week (4–7 days) had a lower incidence of obesity (17.65%) compared to those who spent only 1–3 days per week on vigorous physical activity (38.46%). On the other hand, an increase in the number of days per week spent on moderate physical activity did not lead to a corresponding decrease in the BMI.

**Table 1:** The prevalence of overweight and obesity according to the level of physical activity in Nkonkobe Municipality

	Normal		Obese		Overweight		Underweight		*P*
Variables	*N*	%	*N*	%	*N*	%	*N*	%	
Moderate physical activity									*P* < 0.05
1–3 days/week	25	47.17	13	24.52	13	24.52	2	3.78	
4–7 days/week	20	47.62	18	42.86	4	9.52		0.00	
No physical activity									
	2	10.00	14	70.00	3	15.00	1	5.00	
Vigorous physical activity									*P* < 0.05
1–3 days/week	14	38.46	8	38.46	7	15.38	2	7.69	
4–7 days/week	18	76.47	13	17.65	3	5.88		0.00	

### Alcohol intake and smoking

**Fig. 2:**
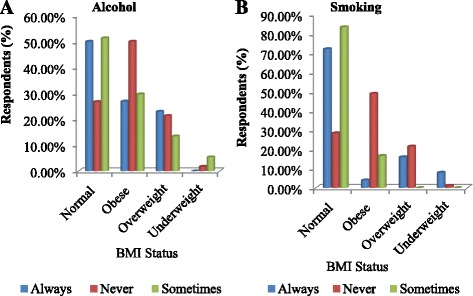
**a** Alcohol intake and **b** smoking habits associated with obesity and overweight in Nkonkobe Municipality

Compared to non-drinkers, people who consumed alcohol regularly had lower odds of being obese, a trend that was also observed among regular smokers (Fig. 2a, b). Obesity was more prevalent among non-smokers and those who never consumed alcohol compared to regular smokers and consumers of alcohol (4.00 and 26.92%), respectively.

### Dietary factors

Close to half (48.48%) of the respondents who consume fast foods always were obese and 30.30% were overweight (Table 2); the prevalence was unexpectedly high among those who never ate fast foods. Obesity prevalence stood at 44.44% among those who consume fruits always, 85.71% among those who never consume vegetables, and very low (11.54%) among regular vegetables consumers (Table 2).

**Table 2:** Dietary habits associated with BMI categories in Nkonkobe Municipality

Variables	Normal		Obese		Overweight		Underweight		*P*
	Normal	%*N*	Normal	%*N*	Normal	%*N*	Normal	%*N*	
Fast foods									
Always	7	21.21	16	48.48	10	30.30		0.00	*P* < 0.05
Sometimes	34	51.52	19	28.79	11	16.67	2	3.03	
Never	5	35.71	8	57.14	1	7.14		0.00	
Vegetables									
Always	6	11.54	6	11.54	15	28.84	25	48.08	*P* < 0.05
Sometimes	10	21.28	12	25.52	12	25.52	13	27.66	
Never	6	42.86	12	85.71	1	7.14	1	7.14	
Fruits									
Always	21	38.89	19	44.44	11	13.89	1	2.78	*P* > 0.05
Sometimes	27	42.86	24	38.10	11	17.46	1	1.59	
Never	1	50.00		0.00	1	50.00		0.00	

### Food preparation factors

**Fig. 3:**
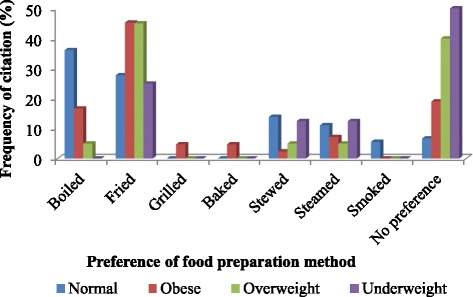
Association of food preparation methods with obesity and overweight in Nkonkobe Municipality

Figure 3 shows the variation of obesity according to preference of food preparation methods among participants. The highest prevalence of obesity and overweight was observed among participants who preferred fried food, followed by those without any special preferences. On the other hand, the prevalence of obesity and overweight was notably low among participants who preferred smoked, baked and grilled foods.

### Co-morbidities

**Fig. 4:**
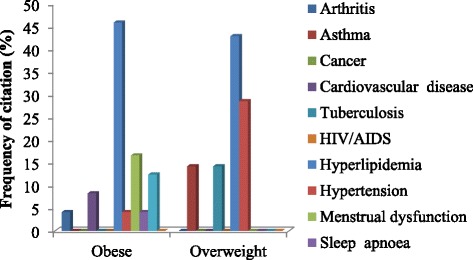
Prevalence of selected co-morbidities associated with obesity and overweight in Nkonkobe Municipality

Arthritis was the most prevalent co-morbidity with incidence of 46.2 and 45.00% among the obese and overweight participants, respectively; this was followed by hypertension (28.00%) in the overweight participants, while the incidence of cancer and tuberculosis was 16.20 and 15.01% among the obese and overweight, respectively (Fig. 4).

## Discussion

The increase in the prevalence of obesity observed worldwide has been linked with the widespread decline in the level of daily physical activity as well as increased availability of energy-dense foods and changing food habits [22]. In this study, the combined prevalence of obesity and overweight in Nkonkobe Municipality was found to be as high as 57%. In sub-Saharan Africa, the prevalence of obesity is highly variable, ranging widely from 1% in Ethiopia to 27% in South Africa [23]. Obesity is a global epidemic that is estimated to rise to about one billion people by 2030 [24], and it is not only limited to the affluent society but also affects the rural people. Poor dietary habits, excessive alcohol intake, cigarette smoking and lack of exercise are some of the lifestyle determinants that have been positively correlated to the development of obesity [25–27]. The findings of this study, however, revealed that lack of physical activity; overindulgence on fast foods, fried food, and fruits and no vegetables in the diet are significant risk factors for obesity in Nkonkobe Municipality.

We observed that the prevalence of overweight and obesity was considerably higher in sedentary people. Sedentary behaviour is defined as any activity that does not increase energy expenditure substantially above a resting level such as sleeping, sitting and lying down [28] and is often assessed as leisure “screen time” such as watching TV, watching videos or using a computer. In this study, we observed an inverse relationship between physical activity, obesity and overweight. Similar results have been reported by other researchers that lack of exercise is a predisposing factor to obesity and overweight especially among adolescents and youths [29–31].

Concordant significant associations between fast food intake, fruits and vegetables, cooking preference, and high BMI was observed. Fast food is associated with higher body mass index, less successful weight-loss maintenance and weight gain. Fast foods reduce the quality of diet and provide unhealthy choices especially among children and adolescents raising their risk of obesity. The result for fast foods is a true reflection of the real situation because the daily intake of fast foods among South Africans is generally high. These findings agree with other researchers who have reported a correlation between fast food and obesity [32, 33]. Interestingly, we also observed a high prevalence in obesity among those who always consumed fruits. According to Xiao et al. [34], the main reason could be that fruit and vegetable intake can be a plausible surrogate for total energy intake. Taking into consideration the low prices of fruits and vegetables, they are generously consumed in every meal with staples and can therefore be positively related to calories from staple intake.

On the other hand, smoking and alcohol intake were not observed as predisposing factors to obesity in the study area. Research has shown that current smokers have a lower mean body mass index (BMI) than never or former smokers, with former smokers having the highest mean BMI [28]. This trend was observed in this study as data revealed that smokers were less prone to obesity and overweight compared to non-smokers. This could be attributed to the fact that smoking suppresses appetite [35] and reduces the activity of lipoprotein lipase responsible for adipose tissue metabolism. Similar trends have been reported by other researchers [36].

The relationship between obesity and alcohol consumption is complex because firstly, alcohol may affect both components of the energy balance equation (energy balance = energy intake − energy expenditure). Secondly, contrary to other energy sources, alcohol does not abide to any specific regulatory mechanisms; hence, alcohol calories are described as “unregulated calories” [37]. In addition, associations between alcohol and obesity are heavily influenced by other factors including patterns and levels of drinking, types of alcoholic drinks consumed, gender, body weight, diet, genes, physical activity levels, and other lifestyle factors [38, 39]. In this study, prevalence of obesity and overweight was higher in subjects not using alcohol than in subjects using alcohol.

The rise in obesity is also associated with an increased risk of non-communicable diseases (NCDs), such as cardiovascular disease, type 2 diabetes and several types of cancer. Obesity is a risk factor for type 2 diabetes, cardiovascular disease, gall bladder disease and some cancers, as well as diseases of the metabolic syndrome [40, 41]. In this study, our data showed that arthritis, hypertension and tuberculosis, asthma, cardiovascular diseases, cancer and sleep apnoea were significantly associated with obesity. This agrees with previous reports by Shukla et al. [17] that obesity is associated with hypertension, diabetes, arthritis, and angina in India, China, Russia and South Africa. Obesity is widely known as a risk factor for both the incidence and progression of osteoarthritis and has a negative effect on outcomes. Loss of at least 10% of body weight, coupled with exercise, is recognized as a cornerstone in the management of obese patients with osteoarthritis and can lead to significant improvement in symptoms, pain relief, physical function and health-related quality of life outcomes [42]. Considering the rural nature of Nkonkobe Municipality, our findings in this study agree with other researchers that physical inactivity, diet, smoking and alcohol are lifestyle factors that predispose rural and urban communities to obesity [1, 3, 4, 18, 21, 43].

## Conclusions

This study has revealed the lifestyle factors and co-morbidities associated with obesity in Nkonkobe Municipality of the Eastern Cape. The combined prevalence of obesity and overweight in this study area was as high as 57%. These findings reveal that lack of physical activity, overindulgence on fast foods, fried food, and fruits and no vegetables in the diet are significant risk factors, while smoking and alcohol intake were not observed as lifestyle factors predisposing to obesity in the study area. Arthritis, hypertension and tuberculosis were significantly associated with obesity; hence, loss of body weight coupled with exercise can be a major cornerstone in the management of obesity with osteoarthritis, which may lead to significant improvement in health-related quality of life in Nkonkobe Municipality.

## Abbreviations

BMI: Body mass index
CVD: Cardiovascular diseases
CDC: Centre for Disease Control and Prevention
MPED-RNA: Medicinal Plants and Economic Development-Research Niche Area
NCDs: Non-communicable diseases
TV: Television

## References

[1] Puoane T, Steyn K, Bradshaw D, Laubscher R, Fourie J, Lambert V, Mbananga N (2002). Obesity in South Africa: the South African demographic and health survey. Obes Res.

[2] Shisana O, Labadarios D, Rehle T, Simbayi L, Zuma K, Dhansay A, Reddy P, Parker W, Hoosain E, Naidoo P, Hongoro C, Mchiza Z, Steyn NP, Dwane N, Makoae M, Maluleke T, Ramlagan S, Zungu N, Evans MG, Jacobs L, Faber M, SANHANES-1 Team. The South African National Health and Nutrition Examination Survey, 2012: SANHANES-1: the health and nutritional status of the nation. 2014 ed. Cape Town: HSRC Press; 2014.

[3] Joubert J, Norman R, Bradshaw D, Goedecke JH, Steyn NP, Puoane T, South African Comparative Risk Assessment Collaborating Group (2007). Estimating the burden of disease attributable to excess body weight in South Africa in 2000. S Afr Med J.

[4] Okop KJ, Levitt N, Puoane T. Factors Associated with Excessive Body Fat in Men and Women: Cross- Sectional Data from Black South Africans Living in a Rural Community and an Urban Township. PLoS ONE. 2015;10(10):e0140153. https://doi.org/10.1371/journal.pone.0140153. 10.1371/journal.pone.0140153 PMC4598161 26447880

[5] Al-Hazzaa HM, Abahussain NA, Al-Sobayel HI, Qahwaji DM, Musaiger AO (2012). Lifestyle factors associated with overweight and obesity among Saudi adolescents. BMC Public Health.

[6] Swinburn BA, Caterson I, Seidell JC, James WPT (2004). Diet, nutrition and the prevention of excess weight gain and obesity. Public Health Nutr.

[7] Munafo MR, Tilling K, Ben-Shlomo Y (2009). Smoking status and body mass index: a longitudinal study. Nicotine Tob Res.

[8] Gallus S, Odone A, Lugo A, Bosetti C, Colombo P, Zuccaro P, La Vecchia C (2013). Overweight and obesity prevalence and determinants in Italy: an update to 2010. Eur J Nutr.

[9] Dare S, Mackay DF, Pell JP (2015). Relationship between smoking and obesity: a cross-sectional study of 499,504 middle-aged adults in the UK general population. Matsuo K, ed. PLoS One.

[10] Rohrer JE, Rohland BM, Denison A, Way A (2005). Frequency of alcohol use and obesity in community medicine patients. BMC Fam Pract.

[11] Suter PM (2005). Is alcohol consumption a risk factor for weight gain and obesity?. Crit Rev Clin Lab Sci.

[12] Sayon-Orea C, Bes-Rastrollo M, Nuñez-Corboda JM, Basterra-Gortari FJ, Beunza JJ, Martinez-Gonzalez MA (2011). Type of alcoholic beverage and incidence of overweight/obesity in a Mediterranean cohort: The SUN project. Nutrition.

[13] Traversy G, Chaput J-P (2015). Alcohol consumption and obesity: an update. Curr Obes Rep.

[14] Pi-Sunyer X (2009). The medical risks of obesity. Postgrad Med.

[15] Guh DP, Zhang W, Bansback N, Amarsi Z, Birmingham CL, Anis AH (2009). The incidence of co-morbidities related to obesity and overweight: a systematic review and meta-analysis. BMC Public Health.

[16] Schienkiewitz A, Mensink GBM, Scheidt-Nave C (2012). Comorbidity of overweight and obesity in a nationally representative sample of German adults aged 18–79 years. BMC Public Health.

[17] Shukla A, Kumar K, Singh A (2014). Association between obesity and selected morbidities: a study of BRICS countries. PLoS One.

[18] Steyn K, Sliwa K, Hawken S, Commerford P, Onen C, Damasceno A, Ounpuu S, Yusuf S (2005). INTERHEART Investigators in Africa. Risk factors associated with myocardial infarction in Africa: The INTERHEART Africa Study. Circulation.

[19] Ondicho ZM, Omondi DO, Onyango AC (2016). Prevalence and socio-demographic factors associated with overweight and obesity among healthcare workers in Kisumu East Sub-County, Kenya. American J Med Med Sci.

[20] Otang WM, Grierson DS, Afolayan AJ (2015). A survey of plants responsible for causing allergic contact dermatitis in the Amathole District, Eastern Cape, South Africa. South African J Bot.

[21] Statistics South Africa. Nkonkobe Local Municipality Integrated Development Plan (IDP) 2015. http://www.statssa.gov.za.

[22] National Nutrition Surveillance Centre (NNSC). The Interrelationship between Obesity, Physical Activity, Nutrition and other Determinates. 8^TH^ position paper by the National Nutrition Surveillance Centre, in partnership with the Health Service Executive (HSE), as part of the HSE Framework for Action on Obesity. 2009; Belfield, Dublin 4, Ireland.

[23] Micklesfield LK, Lambert EV, Hume DJ, Chantler S, Pienaar PR, Dickie K, Goedecke JH, Puoane T (2013). Socio-cultural, environmental and behavioural determinants of obesity in black South African women. Cardiovasc J Africa.

[24] World Health Statistics. WHO Statistical Information System (WHOSIS). Global Health Observatory. 2008. http://www.who.int.

[25] Yach D, Stuckler D, Brownell KD (2006). Epidemiologic and economic consequences of the global epidemics of obesity and diabetes. Nat Med.

[26] Barry D, Clarke M, Petry NM (2009). Obesity and its relationship to addictions: is overeating a form of addictive behavior?. Am J Addict.

[27] Rabaeus M, Salen P, de Lorgeril M (2013). Is it smoking or related lifestyle variables that increase metabolic syndrome risk?. BMC Med.

[28] Kaufman A, Augustson EM, Patrick H. Unraveling the Relationship between Smoking and Weight: The Role of Sedentary Behavior. J Obes. 2012; Article ID 735465:11. doi:10.1155/2012/735465. 10.1155/2012/735465 PMC3180774 21961058

[29] Owen N, Bauman A, Brown W (2009). Too much sitting: a novel and important predictor of chronic disease risk. The British J Sport Med.

[30] Stankov I, Olds T, Cargo M (2012). Overweight and obese adolescents: what turns them off physical activity?. Int J Behav Nutr Phys Act.

[31] Labban L (2014). The association between physical activity, overweight and obesity among Syrian university students. Saudi J Sports Med.

[32] Monteiro CA (2009). Nutrition and health. The issue is not food, nor nutrients, so much as processing. Public Health Nutr.

[33] De Vogli R, Kouvonenb A, Gimeno D (2014). The influence of market deregulation on fast food consumption and body mass index: a cross-national time series analysis. Bull World Health Organ.

[34] Xiao Y, Zhao N, Wang H, Zhang J, He Q, Su D, Zhao M, Wang L, Zhang X, Gong W, Hu R, Yu M, Ding G, Cong L, Ye Z (2013). Association between socioeconomic status and obesity in a Chinese adult population. BMC Public Health.

[35] Chiolero A, Jacot-Sadowski I, Faeh D, Paccaud F, Cornuz J (2007). Association of cigarettes smoked daily with obesity in a general adult population. Obesity.

[36] Morgenstern M, Isensee B, Hanewinkel R (2010). Gender, smoking and weight concerns: relationship to self-reported body mass index (BMI). Przegl Lek.

[37] Gatineau M, Dent M (2012). Obesity and alcohol: an overview.

[38] Shelton NJ, Knott CS (2013). Association between alcohol calorie intake and overweight and obesity in English adults. Am J Public Health.

[39] Brunner EJ, Chandola T, Marmot MG (2007). Prospective effect of job strain on general and central obesity in the Whitehall II study. Am J Epidemiol.

[40] Liu WJ, Rong L, Liu AL, Lin D, Qing C (2010). Prevalence and association between obesity and metabolic syndrome among Chinese elementary school children: a school-based survey. BMC Public Health.

[41] Bliddal H, Leeds AR, Christensen R (2014). Osteoarthritis, obesity and weight loss: evidence, hypotheses and horizons—a scoping review. Obes Rev.

[42] Mungreiphy NK, Kapoor S, Sinha R. Association between BMI, blood pressure, and age: study among Tangkhul Naga Tribal Males of Northeast India. J Anthropol. 2011; Article ID 748147:6.

[43] Van Zyl S, Van der Merwe LJ, Walsh CM, Groenewald AJ, Van Rooyen FC. Risk-factor profiles for chronic diseases of lifestyle and metabolic syndrome in an urban and rural setting in South Africa. Afr J Prm Health Care Fam Med. 2012;4(1):1–10.

